# 3D Surface Profile and Color Stability of Tooth Colored Filling Materials after Bleaching

**DOI:** 10.1155/2015/327289

**Published:** 2015-10-19

**Authors:** Bryant Anthony Irawan, Stacey Natalie Irawan, Sam'an Malik Masudi, Ninin Sukminingrum, Mohammad Khursheed Alam

**Affiliations:** ^1^Stanford University, 450 Serra Mall, Stanford, CA 94305, USA; ^2^School of Dental Sciences, Universiti Sains Malaysia, 16150 Kubang Kerian, Kelantan, Malaysia

## Abstract

This study aims to evaluate the effects of vital tooth bleaching with carbamide peroxide home bleaching and in-office bleaching on the color stability and 3D surface profile of dental restorative filling materials. Thirty discs (*n* = 30) measure 6 mm in diameter and 2 mm thick for each of three restorative materials. These are nanofilled composite Filtek Z350 XT, the submicron composite Estelite Σ Quick, and nanofilled glass ionomer Ketac N100 nanoionomer and were fabricated in shade A2. Each group was further divided into three subgroups (*n* = 10): subgroup A (Opalescence PF), subgroup B (Opalescence Boost in-office bleaching), and subgroup C (distilled water) serving as control. Samples were bleached according to the manufacturer's instructions for a period of two weeks. The Commission Internationale de L'Eclairage (CIE *L* ^*^, *a* ^*^, *b* ^*^) system was chosen for image processing, while 3D surface profile was tested with atomic force microscopy (AFM). Statistical analyses were performed with the Mann-Whitney tests and Krusal-Wallis with a *P* value of ≤0.05. The three restorative materials showed significant color changes (Δ*E*); *P* ≤ 0.05. In diminishing order, the mean color changes recorded were Estelite Σ (3.82 ± 1.6) > Ketac Nano (2.97 ± 1.2) > Filtek Z350 XT (2.25 ± 1.0). However, none of the tested materials showed statistically significant changes in surface roughness; *P* > 0.05.

## 1. Introduction

Home bleaching has gained considerable acceptance among dentists and patients as a simple, effective, and safe procedure to lighten discolored teeth. Since its introduction by Haywood and Heymann in 1989 [1], tooth whitening has become one of the most popular esthetic procedures offered by dentists. There are several types of bleaching methods, but all of them share the common principle of the degrading of peroxides from hydrogen peroxide or its compounds such as carbamide peroxide (CP) into unstable free radicals. These radicals are further broken down into large pigmented molecules through either oxidation or reduction reaction [2].

The oxidation or reduction process changes the chemical structure of the interacting organic substances of the tooth, which results in the change in color [2]. Furthermore, Maleknejad et al. [3] reported an increase in the diameter of dentinal tubules at a concentration of 45% CP as a result of different intracoronal tooth-bleaching procedures. They also reported alterations in the mineral content of the dentin.

Bleaching methods include nonvital bleaching, in-office professional bleaching, and home bleaching. Tray-delivered home bleaching uses a relatively low concentration of whitening gel, which is applied to the teeth by means of a custom fabricated tray, which is worn at night for the duration of at least two weeks [4].

Considerable research has been carried out to identify the effects of bleaching on the tooth surface and dental restorative materials. Jacob and Dhanya Kumar [5] reported that bleaching with CP might affect the marginal leakage of resin composite restorations; however, amalgam restorations were not adversely affected* in vitro*. The CP agents were observed to have a profound influence on the color behavior of tooth colored restorations [6] or perhaps even cause deterioration [7]. Interaction between the bleaching agents and the restorative material may result in noticeable color change especially if the color closely matched the tooth structure before bleaching [8]. Thus, as result of bleaching, the end result could be an esthetic failure due to an incorrect color match. As a result, replacement of the existing restoration may be required. Studies have shown that the color stability of tooth colored restorative materials depends on the type of material [8].

Composite resin and glass ionomers are the most popular dental restorative materials. They offer superior esthetics, require minimal tooth preparation, and are widely used for anterior and posterior restorations. Recently a nanofilled resin composite and glass ionomer were introduced and exhibit a high initial polish while retaining this over time combined with excellent physical properties [10].

In composite resin technology, particle size and quantity are crucial when determining how to best utilize the restorative materials. Alteration of the filler component remains the most significant development in the evolution of composite resins [11]. The filler particle size, distribution, and the quantity incorporated dramatically influence the mechanical properties and clinical success of composite resins [12].

Filtek Z350 XT is nanohybrid resin composite material. To moderate the shrinkage, PEGDMA has been substituted for a portion of the TEGDMA resin. The fillers are a combination of nonagglomerated/nonaggregated 20 nm silica filler, nonagglomerated/nonaggregated 4 to 11 nm zirconia filler, and aggregated zirconia/silica cluster filler (comprised of 20 nm silica and 4 to 11 nm zirconia particles). The nanocomposites have an average cluster particle size of 0.6 to 10 microns while the inorganic filler loading is about 72.5% by weight (55.6% by volume).

Estelite Σ Quick is microhybrid composite resin which contains BisGMA and triethylene glycol dimethacrylate (TEGDMA) with filler size of 0.1–0.3 *μ*m and filler loading is 82% by weight or 71% by volume.

Generally, glass ionomer restoratives can contain a broad range of particle sizes. Filler particle size can influence strength, optical properties, and abrasion resistance. By using bonded nanofillers and nanocluster fillers, along with FAS glass, nanoionomer restorative has improved esthetics and low wear yet still provides the benefits of glass ionomer chemistry, such as fluoride release. Overall, nanoionomer restorative exhibits impressive surface characteristics.

It has been reported that bleaching agents reduce the microhardness of enamel and promote an increase in surface roughness [13]. There exists a significant and positive correlation between surface roughness and bacterial adhesion [14]. Roughness has a major impact on esthetic appearance, discoloration of restorations, plaque accumulation, secondary caries, and gingival irritation [15]. Interaction between bleaching agents and the restorative material may result in noticeable color change especially if the color matched the tooth structure closely before bleaching.

On the other hand, studies have also shown that the effect of bleaching agents is minimal with regard to roughening of composite resin surfaces and that they do not perceptibly change the shade of tooth colored materials [16]. However, another study concluded that nanofilled resin composites are more resistant and are preferred as a restorative material when bleaching treatment is indicated [17].

In this study, samples were analyzed using the CIELAB color technique. Standard Commission Internationale de L'Eclairage (CIELAB) is color system that assesses chromatic differences in colorimetry. The use of digital cameras to capture accurate color in dentistry is advantageous in the color replication process for any craniofacial prosthesis, given the potential to capture the polychromatic color of the structure, as well as form, texture, and perceived translucency [9]. A color difference of Δ*E* = 2 in the CIELAB color systems was detectable by the human eye under uniformly controlled conditions [18].

There is limited data on the effects of bleaching agents on microhybrid and nanofilled composite resins, as well as the new nanofilled glass ionomers. There is also scant knowledge concerning the effect of the in-office and home bleaching systems on these latest developments. Current study hypothesized that there are no differences between in-office and home bleaching systems on the color stability and 3D surface profile of tooth colored restorative materials. The aim of this* in vitro* study was to evaluate the effect of home versus in-office bleaching systems on the color stability and 3D surface profile of tooth colored restorative materials.

## 2. Materials and Methods

The Ethics Committee of the Universiti Sains Malaysia (Kelantan, Malaysia) reviewed and approved the research project. This* in vitro* study evaluated the color stability and 3D surface profile of three tooth colored restorative materials after bleaching. Two commercially available nanohybrid and microhybrid, Bis GMA-based resin composites and one nanoionomer, all with an A2 shade, were used in the present study (Table 1). Properties of the research materials were presented in Table 2. Samples were fabricated in 2 mm thick plexiglass with a circular opening of 6 mm. After insertion of the test material polyethylene was applied and the material pressed down with glass slabs. Excess material was removed. A total of 90 samples were prepared, thirty samples (*n* = 30) for each test material (Group I (*n* = 30): Filtek Z350 XT nanohybrid composite 1, Group II (*n* = 30): Estelite Σ microhybrid composite 2, and Group III (*n* = 30): Ketac N100 nanoionomer cement 1).

All samples were light-cured from the top and bottom using an Elipar Freelight 2^1^ according to the manufacturer's instructions with an output of 1000 mW/cm^2^ and wave length of emitted light of 430–480 nm. The nanoionomer cement after placement was light-cured for 20 s, while the resin composite materials were placed incrementally into the circular opening and light-cured for 20 seconds each increment.

All samples were then polished using Sof-Lex 1 from coarse (55 *μ*m) to medium (40 *μ*m) to fine (24 *μ*m) and ultrafine (8 *μ*m), using a mandrel and a slow-speed hand piece. Polishing was carried out without water for 10 seconds per disk. An effort was made to standardize downward force and number of strokes for each disk. After polishing, the samples were cleaned ultrasonically using a Sonica 2200 ETH 3 for 5 minutes and then stored in distilled water at 37°C for 24 hours prior to the bleaching treatment.

Each group was further divided into three subgroups of 10 specimens each (*n* = 10). The specimens were subjected to bleaching agents following the manufacturers' instructions. Samples in subgroup A (control group) were not bleached but stored in a vibrating distilled water bath 4 for 14 days at 37°C.

Samples in subgroup B were subjected to Opalescence home bleaching PF, a 20% CP 5, for four hours per day for 14 days according to the instructions of the manufacturer.

Subgroup C was treated with Opalescence in-office bleaching chair-side Whitening Boost, a 40% CP 5, for 40 minutes (2 × 20 minutes) per day for a total of 5 days. The mixing procedure of the Opalescence Boost bleaching gel and application of a 0.5–1.0 mm thick layer of gel on the sample was carried out according to the manufacturer's instructions.

In subgroups B and C, prior to each bleaching procedure, the samples were removed from the distilled water bath and air-dried with an oil-free air jet spray for 60 seconds. The bleaching agent was applied on one surface using a microbrush 6 and left in place for the duration suggested by the manufacturer. After each bleaching procedure, the samples were washed with an air/water spray for 60 seconds before they were stored again in distilled water at 37°C until the next bleaching session.

Duration time of bleaching for each subgroup followed the manufacturer's instructions. The bleaching protocol was carried out daily for two weeks.

### 2.1. Analysis of Color Stability

**Figure 1:**
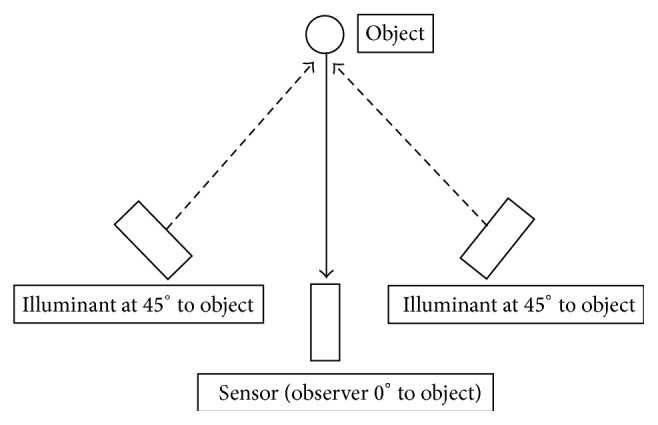
Schematic view of the experimental set-up [9].

The samples were placed on a neutral grey card and photos were taken with a Nikon D200 digital camera in a darkened room with the main source of light coming from two Phillips F15TS 15 watt bulbs at 45 degrees (Figure 1). CIELAB color values were analyzed from digital raw images taken from the samples using software Photoshop CS3 Ver 10.0. All specimens were measured twice and the average values were calculated. The calculations of the color variations (Δ*E*) were made between two color positions.

The CIE LAB-based color difference formula, introduced in 1976 and recommended by the International Commission on Illumination [9], defines a color space (*L* ^*∗*^ *a* ^*∗*^ *b* ^*∗*^) in which *L* ^*∗*^ represents lightness, *a* ^*∗*^ represents the chromaticity coordinate for red-green (C*a* ^*∗*^Z red direction; K*a* ^*∗*^Z green direction), and *b* ^*∗*^ represents the chromaticity coordinate for yellow-blue (C*b* ^*∗*^Z yellow direction; K*b* ^*∗*^Z blue direction). The magnitude of total color difference (between baseline and after bleaching measurements) is represented by a single number Δ*E* (Commission Internationale de L'Eclairage, 1979):

(1) $$\begin{array}{c} \Delta E={\left[{\left(\Delta L^{*}\right)}^{2}+{\left(\Delta a^{*}\right)}^{2}+{\left(\Delta b^{*}\right)}^{2}\right]}^{1/2}, \end{array}$$

where Δ*L* ^*∗*^, Δ*a* ^*∗*^, and Δ*b* ^*∗*^ are the respective difference between the measured and predicted CIE LAB values of the shade.

### 2.2. 3D Surface Profile Measurements

**Figure 2:**
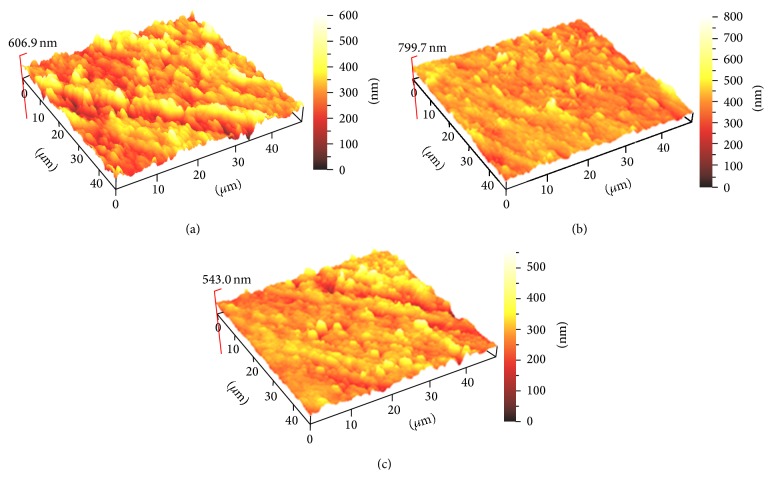
The AFM-3D images of Z350 XT surface roughness: (a) Z350 XT without bleaching, (b) Z350 XT with home bleaching, and (c) Z350 XT with in-office bleaching.

**Figure 3:**
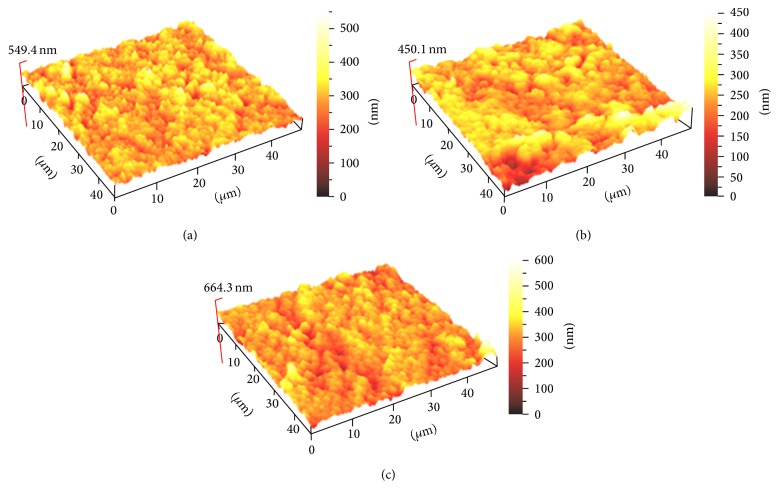
The AFM-3D images of Ketac N100 surface roughness: (a) Ketac N100 without bleaching, (b) Ketac N100 with home bleaching, and (c) Ketac N100 with in-office bleaching.

**Figure 4:**
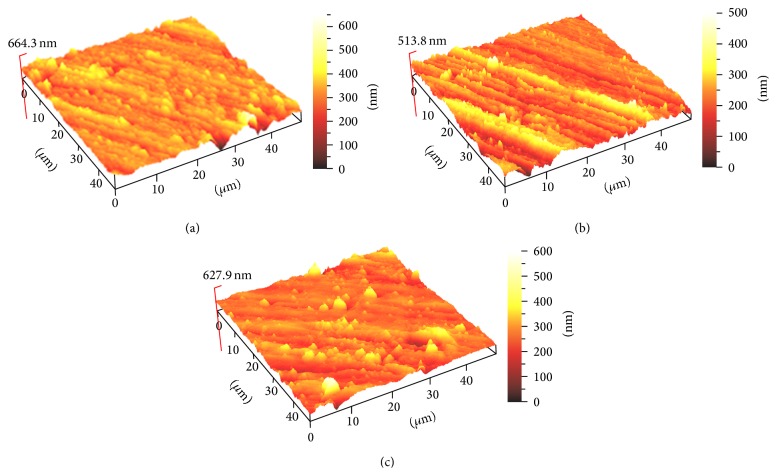
The AFM-3D images of Estelite Σ Quick surface roughness: (a) Estelite Σ without bleaching, (b) Estelite Σ with home bleaching, and (c) Estelite Σ with in-office bleaching.

Six samples from each group were subjected to 3D surface profile evaluation using atomic force microscopy (AFM)^7^. The mean 3D surface profile was assessed with a contact mode. Five different randomly selected areas were scanned with an area of 40 × 40 *μ*m and resolution of 512 × 512 pixels to obtain surface roughness values (Ra). Ra analysis was done by ScanAtomic SPM control software. Then, three-dimensional (3D) images with 10 × 10 *μ*m sizes were acquired for each group of materials (Figures 2, 3, and 4).

The data collected were analyzed using SPSS version 16.0. All of the statistical analysis was conducted at a significance level of *P* < 0.05 using the Mann-Whitney and Kruskal-Wallis test.

## 3. Results

Δ*E* is compared within the groups and between the subgroups.

Table 3 shows a comparison of Δ*E* value between restorative materials when treated with a different bleaching agent. Filtek Z350 XT has a higher mean color change as a result of in-office bleaching compared to home bleaching. In contrast, Estelite Sigma Quick and Ketac N100 demonstrated a higher Δ*E* value after home bleaching compared to in-office chair-side bleaching.

Table 4 shows the comparison of mean color change of the restorative materials between the two bleaching agents. The mean color change of Ketac N100 was the highest, followed by Estelite Sigma Quick for both in-office and home bleaching agents. Filtek Z350 XT showed the least color changes.

Table 5 presents the roughness numbers (Ra) of the restorative materials that were bleached. Statistically insignificant changes were found in roughness for all three materials tested after 14 days of bleaching with 10 and 20% CP compared to the control group.

## 4. Discussion

Home bleaching and in-office bleaching are popular treatment modalities that are attractive to dentists and patients, as they constitute a simple, safe, and effective procedure to lighten discolored teeth. However, preexisting Classes I, II III, IV, and V tooth colored restorations may be affected by the bleaching gels. Bleaching agents may result in a color change of a restoration that may be perceived by the patient and determined to be unacceptable. If a restorative material has a perfect color match with the surrounding tooth before bleaching, this may no longer be the case after bleaching when the teeth have become lighter and brighter as a result of the CP treatment. Within the limits of this study, it was observed that even low concentrations of bleaching agents had an effect on the color of restorative materials.

Considering the active ingredients available for vital tooth bleaching, carbamide and hydrogen peroxide are the most commonly used agents for different bleaching modalities. Carbamide peroxide degrades into approximately one-third of hydrogen peroxide and two-thirds of urea [19]. The free radicals that are formed eventually combine to form molecular oxygen and water. Some aspects of this chemical process might accelerate the hydrolytic degradation of restorative materials, as described by Söderholm et al. [20]. Chemical softening of the restorative materials might also occur if the bleaching products have a high concentration of hydrogen peroxide [21].

GIC's color change is due to its polyacid content, while the composite color changes are influenced by many factors such as resin shades, the chemical activator, initiator, and inhibitor. The resin component was determined to be the source of discoloration [18].

The A2 shade was chosen for composite materials to minimize the effect of shade variation. Two marketed bleaching systems that differed with respect to peroxide concentration and regimen were compared: Ultradent Opalescence Boost (40% hydrogen peroxide) for in-office bleaching and Ultradent Opalescence PF (20% hydrogen peroxide) for home bleaching. Control specimens were used against which the effects of bleaching were compared.

The color of dental esthetic restorative materials is routinely measured with commercial DSLR cameras and appropriate calibration protocols. In assessing chromatic differences, CIELAB was used in this study.

The lightening of the specimens was depicted as an increase in *L* while the actual hue-chroma change was demonstrated in changes in *A* or *B*. The amount of discoloration after a given period was represented by the color difference value (Δ*E*). The accepted change caused by these bleaching preparations produces a Δ*E* value of 2, which is less than that of visual perception [22]. Thus, the human eye cannot detect a change in color of a material that has undergone a change of less than Δ*E* of 2 [22]. Therefore, a minimum difference of 2 can be used as criteria for the comparison of color changes in the restorative materials [23]. Wee et al. [9] concluded that perceptible color differences range from a Δ*E* of 1 and 2 in* in vitro* studies to 3.7 in an* in vivo* study, while acceptable color differences range from a Δ*E* of 2.72 and 3.3 in* in vitro* studies to 6.8 in an* in vivo* study. In another study, Yalcin and Gurgan [24] reported that bleaching regimens may also cause a change in gloss of restorative materials.

Among the materials tested, Estelite Sigma Quick showed the largest color change (Δ*E* = 3.8), followed by Ketac N100 (Δ*E* = 3.1) and Filtek Z-350 XT (Δ*E* = 2.2). This can be explained by the degradation of metal polyacrylate salts. Color changes of composites may be influenced by the differences in resin shades, the chemical activator, initiator, and inhibitor, polymer, type and quantity of filler, oxidation of C=C double bonds, resin thickness, or storage methods of specimens during observation [24]. Filtek Z-350 showed the least color change followed by Ketac N100. This may be attributed to the amount of nanofiller particles present in the composite resin [7]. Canay and Çehreli [8] have also reported that the change in color is associated with the matrix content, the amount of filler, and the type of filler material.

The size and morphology of filler particles influence the mechanical and physical properties while nanoparticles and clusters in the nanofilled materials improved it [25]. Higher discoloration of the Estelite Sigma Quick may be due to the greater volume of the resin composite matrix when compared with Filtek Z-350 [26]. The bleaching agents may also cause a decline of silica and silicon content, indicating erosion of the resin composite material [27]. In addition, the color changes of composites were also influenced by the differences in curing conditions, background colors for color measuring, color measuring methods, type of color measuring instruments, and observation methods [24].

From the results we also determined that the mean color change of all tested restorative materials was greater for home bleaching than in-office bleaching. This may be due to the longer application time, in spite of the fact that the concentration of hydrogen peroxide is lower for home bleaching agents. According to Meireles et al. [23], lower carbamide peroxide concentrations were more effective in the first week of their study. It appears that total contact time of bleaching gels is more important than the concentration.

Another study showed that higher concentrations of bleaching agents achieve the same postbleaching result as lower concentration. However, the higher concentration accomplished the whitening result more quickly [28]. The results of our study suggest that the final color change does not depend on the concentration of the bleaching agent but rather on the application time.

The AFM method senses any irregularities on the surface of the specimen and in this study no significant differences between the materials were recorded. This is in agreement with findings of Silva et al. [29]. However, our data contradicts Hafez et al. [30] who reported an increased surface roughness of composites resin, which they determined depending on the bleaching agent as well as the type and shade of composite material tested.

Generally, the 3D surface profile that was recorded had a reading of below 0.2 *μ*m or 200 nm. It has been reported that Ra above 0.2 nm results in an increase in plaque accumulation resulting in a higher risk for caries and periodontal disease [31]. According to Chung [32], when Ra was lower than 1 *μ*m, the surfaces were visibly smooth. Therefore, all composites surfaces evaluated after bleaching demonstrated a smooth surface, which from a clinical point of view is favorable as it reduces plaque accumulation.

As was reported here, even low concentrations of bleaching agents had an effect on the color of restorative materials. Patients should be informed that existing restorations may not match their natural teeth after bleaching and replacement may be required for esthetic reasons. However, this has to be further investigated with* in vitro* studies evaluating the effects of saliva as well as controlled clinical trials.

This study evaluated the effect of home versus in-office bleaching systems on the color stability (CIELAB) and 3D surface profile (AFM) of nanofilled tooth colored restorative materials. This combination study was the advanced and different method to previous bleaching studies.

*Limitations of Study.* The study was carried out to compare color stability and surface roughness of tooth colored filling material* in vitro*. Oral simulating condition cannot be achieved, especially saliva.

## 5. Conclusion

Submicron filled resin composites showed the highest color changes followed by Ketac nanoionomer after bleaching. The nanofilled composite was found to be highly stable in terms of color. Nanofilled composite and a glass ionomer showed better color stability compared to a microfilled tooth colored material.

Based on the result of this study, it can be concluded that 20% CP home bleaching and 40% CP in-office bleaching agents did not cause changes in surface roughness of the three tested composites. The AFM evaluation of surface roughness observed in the 3D images proved to be an effective technique. Nanohybrid resin composite, microhybrid composite resins, and nanoionomer bleached with 20 or 40% CP bleaching agents resulted in the same 3D surface profile.

*Clinical Significance in Dentistry.* Dental practitioner should make sure that their patients with dental restorations (especially those with polymer content) are aware of the changes that may occur during whitening, as well as the possibility that their bleached restorations may need to be polished or replaced at the end of the treatment.

As was reported here, even low concentrations of bleaching agents had an effect on the color of restorative materials. Patients should be informed that existing restorations may not match their natural teeth after bleaching and replacement may be required for esthetic reasons.

For dental society, this result will give information to the dental practitioner regarding the effect of home and in-office bleaching to the new available tooth colored filling materials. This information is important for dental practitioner to make decision on material to be chosen in tooth whitening treatment, for the benefit of patients.

*Recommendations.* Further clinical study should be conducted on color stability of tooth colored filling material after tooth whitening evaluating the effects of saliva and other oral environments. Controlled clinical trials are necessary to determine any clinical implication.

## Figures and Tables

**Table 1:** Composites resin and bleaching agents tested.

Materials	Composition	Manufacturer	Batch number
Filtek Z350 XT (nanohybrid composite resin)	BisGMA, Bis-EMA, UDMA, and TEGDMA Filler size of 5–20 nm Filler loading is 78.5% by weight or 58.5% by volume	3M ESPE, St. Paul, MN, USA	N179865

Estelite Σ Quick (microhybrid composite resin)	BisGMA and triethylene glycol dimethacrylate (TEGDMA) Filler size of 0.1–0.3 *μ*m Filler loading is 82% by weight or 71% by volume	Tokuyama Dental, Tokyo, Japan	E542

Ketac N100 (resin-modified nanoglass ionomer)	Based on the methacrylate-modified polyalkenoic acid Deionized water Methacrylate: blend including HEMA Polyalkenoic acid: VBCP Glass: acid-reactive FAS glass, nanoparticles, and nanoclusters	3M ESPE, St. Paul, MN, USA	N389644

Opalescence home bleaching: Opalescence PF	20% carbamide peroxide Potassium nitrate 0.11% fluoride ions	Ultradent Products Inc., South Jordan, UT, USA	

Opalescence in-office bleaching chair-side Whitening: Opalescence Boost	40% hydrogen peroxide	Ultradent Products Inc., South Jordan, UT, USA	

Bis-GMA: bisphenol-glycidyl methacrylate; BIS-EMA: ethoxylated bisphenol A glycol dimethacrylate: UDMA: urethane dimethacrylate: TEGDMA: triethylene glycol dimethacrylate: HEMA: hydroxy ethyl methacrylate; VBCP: vitrebond copolymer; FAS: fluoroaluminosilicate.

**Table 2:** Properties of research materials.

Material	Type of material	Properties
Filtek Z350 XT	Nanohybrid composite resin	(i) Nanofiller improves compression strength and/or hardness, flexural strength, elastic modulus, coefficient of thermal expansion, water absorption, and wear resistance (ii) Optimizing the adhesion of restorative biomaterials to the mineralized hard tissues of the tooth is a decisive factor enhancing the mechanical strength, marginal adaptation, and seal, while improving the reliability and longevity of the adhesive restoration

Estelite Σ Quick	Microhybrid composite resin	(i) Outstanding polishability (ii) Wide shade matching range (chameleon effect) (iii) High gloss retention over time (chameleon effect) (iv) High wear resistance (v) Low shrinkage (vi) Good radiopacity

Ketac N100	Resin-modified nanoglass ionomer	(i) Nanoionomer is the first paste/paste, resin-modified glass ionomer developed with nanotechnology (ii) Using fluoroaluminosilicate (FAS) technology (iii) Exhibiting impressive surface characteristics (iv) High fluoride release (v) Improved wear resistance (vi) Radiopaque (vii) Light cure on demand

Opalescence PF	Home bleaching	(i) Low concentration of 20% carbamide peroxide, potassium nitrate, and fluoride ions

Opalescence Boost	In-office bleaching chair-side Whitening	(i) High concentration of 40% hydrogen peroxide

**Table 3:** Comparison of Δ*E* between 3 different tooth colored restorative materials after bleaching (between restorative materials).

Restorative material	Bleaching agent	Mean ± SD	*P* value
Filtek Z350 XT	20% CP HB	2.2 ± 1.02	0.0390^*^
	40% CP OB	2.6 ± 1.1	

Estelite Sigma Quick	20% CP HB	3.7 ± 1.5	0.0020^*^
	40% CP OB	3.0 ± 1.2	

Ketac N100	20% CP HB	3.1 ± 1.2	0.0160^*^
	40% CP OB	2.7 ± 1.2	

Mann-Whitney test; ^*∗*^ *P* value < 0.05 is significant; CP: carbamide peroxide.

**Table 4:** Comparison of Δ*E* value of the tooth colored restorative materials after bleaching (between the bleaching agents).

Bleaching agent	Restorative material	Mean ± SD	*P* value
Home bleaching 20% CP	Filtek Z350 XT	2.2 ± 1.0	0.0001^*^
	Estelite Σ Quick	3.7 ± 1.5	
	Ketac N100	3.1 ± 1.2	

In-office bleaching 40% CP	Filtek Z350 XT	2.6 ± 1.1	0.0001^*^
	Estelite Σ Quick	3.0 ± 1.2	
	Ketac N100	2.7 ± 1.2	

Mann-Whitney test; ^*∗*^ *P* value < 0.05 is significant; CP: carbamide peroxide.

**Table 5:** Median roughness number (Ra, nm) and interquartile range of the three tested composite resins after bleaching with home and in-office bleaching agent.

Material	Control *n* = 10 Median (IqR)	20% CP *n* = 10 Median (IqR)	40% CP *n* = 10 Median (IqR)	*P* value
Filtek Z350XT	73.87 (13.73)	71.73 (10.47)	68.43 (14.25)	0.537
Estelite Σ Quick	77.86 (17.55)	75.26 (11.76)	74.87 (15.84)	0.491
Ketac N100	72.49 (10.31)	72.85 (12.36)	70.22 (13.79)	0.635

Kruskal-Wallis test; *P* value < 0.05 is significant; IqR: interquartile range; CP: carbamide peroxide.

## Endnotes

1. 3M ESPE, Minneapolis, MN, USA

2. Tokuyama Dental, Tokyo, Japan

3. Tuttnauer, Breda, Netherlands

4. SW23, Julabo, Seelbach, Germany

5. Ultradent Products Inc., South Jordan, UT, USA

6. Kerr Corporation, Orange, CA, USA

7. Ambios Technology, Santa Cruz, CA, USA.
